# Pregorexia: a systematic review and meta-analysis on the constructs of body image dissatisfaction and eating disturbances by gestational age in the peripartum

**DOI:** 10.1007/s40519-023-01595-8

**Published:** 2023-08-01

**Authors:** Livio Tarchi, Giuseppe Pierpaolo Merola, Giulia Selvi, Eleonora Caprara, Vincenzo Pecoraro, Emanuele Cassioli, Eleonora Rossi, Felice Petraglia, Valdo Ricca, Giovanni Castellini

**Affiliations:** 1 Psychiatry Unit, Department of Health Sciences, University of Florence, AOU Careggi, Viale Della Maternità, Padiglione 8B, 50121 Florence, FI Italy; 2 Obstetrics and Gynecology Unit, Department of Experimental and Clinical Biomedical Sciences, University of Florence, Florence, Italy

**Keywords:** Eating psychopathology, Body image, Feeding and eating disorders, Pregnancy, Postpartum

## Abstract

**Purpose:**

Pregorexia is a phenomenon posited to occur in the peripartum, characterized by an attempt to counter pregnancy’s physiological changes in body shape through reduced calorie intake or increased physical activity.

**Methods:**

In this pre-registered systematic review and meta-analysis, body image dissatisfaction and eating psychopathology in the peripartum according to gestational age were formally assessed. PubMed was searched up to May 2023. Selection criteria were represented by studies on body image concerns or eating psychopathology in the peripartum (up to 1 year after delivery). The population needed to include women from the general population or among patients with a history of (or current) eating disorder. For the meta-analysis, 17 studies were included: 10 for body image dissatisfaction (2625 individuals overall), 7 for eating behaviors (2551 individuals overall). The interplay between body image and the following themes was examined in depth: the adoption of breastfeeding, peripartum depression, sociocultural influences on body image, sexual disturbances, experiencing or reporting an altered food intake.

**Results:**

Progressive dissatisfaction with body image during pregnancy by gestational age was observed, stably elevated for at least 12 months postpartum. Eating psychopathology was observed as elevated only at 12 months in the postpartum, but not during pregnancy.

**Discussion:**

The current work offers normative values of body image satisfaction and eating psychopathology in the peripartum by gestational age. The relevance of current results was discussed, in order to inform both current clinical practice and future public policies.

**Level of Evidence:**

Level I—Evidence obtained from: systematic reviews and meta-analyses.

**Supplementary Information:**

The online version contains supplementary material available at 10.1007/s40519-023-01595-8.

## Introduction

“Pregorexia” is a condition posited to occur during pregnancy. It is characterized by attempts to reduce caloric intake and increase physical activity in order to counter pregnancy’s physiological and undesired changes to body shape in women [1, 2]. These behaviors and phenomena could be under the influence of a specific eating psychopathology, fueling compensatory behaviors in light of bodily distress and body image distortions. Pregorexia has been brought to the attention of the general public and scientific literature in recent years, following early reports and qualitative studies on the topic [2]. Both pregnancy and the postpartum period are critical time frames of vulnerability in women’s life [3, 4], during which psychopathologies can either be induced or relapse. In particular, the scientific literature has focused on new onsets or relapses during the peripartum for mood disorders or psychotic episodes [5, 6]. Nonetheless, increased awareness about body image dissatisfaction and pathological eating behaviors allowed clinicians to also recognize the burden of eating disorders (EDs) [7], during the same period of life in women [8], improving diagnosis and treatment accordingly. Moreover, with improved diagnosis and treatment, the identification of reproductive health concerns has also increased for chronic ED patients [9, 10]. For instance, complications are known to arise and impact the pregnancy of ED patients more frequently than the general population [11, 12]. Thus, investigating the effects that an objectively changing body could have on the psychopathologies of these patients is of clinical and scientific interest, not only from a psychiatric point of view but from an obstetrical perspective as well. In fact, there is ample evidence that food restriction and relevant calories deficit pose a concrete and real threat to patients during pregnancy, delivery and postpartum care [9, 13], while also predisposing to negative outcomes in the offspring [14].

Social pressures and gendered discourses on femininity may either reinforce or moderate a pre-existing undue influence of body weight and shape on the opinion of oneself during or after pregnancy [15, 16]. At the present day, conflicting evidence exists whether these effects are indeed protective or predisposing for eating disturbances during pregnancy, as well as for body weight or shape preoccupations, that could drive significant food restriction behaviors in a critical period of fetal development and maternal life. Therefore, a systematic assessment of this phenomenon is warranted, as food restriction during pregnancy has the potential to dramatically worsen the course of an otherwise physiological pregnancy from a clinical point of view [13]. On the other hand, excessive weight gain or disordered eating have also been correlated with worsened fetal conditions at birth [17, 18].

The common theoretical basis for all EDs is conceptualized as a disturbance in body image [19, 20]. To what extent the changing body experienced during pregnancy can represent an aggravating factor in predisposed individuals, or whether women are diagnosed during pregnancy for a pre-existing condition, is currently debated [21]. In other words, following a classical diathesis-stress model [22, 23], it can be posited that pregnancy elevates stress related to body image, and personal vulnerability interacts with the gestational experience defining an individual stress response, informed by environmental, cognitive and social factors. The present work thus analytically reviews and discusses the present evidence on the topic, offering quantitative evidence in favor of the hypothesis that pregnancy itself represents a decisive point in life for psychological and psychopathological factors, informed and influenced by affective, cognitive, and social determinants.

### Aims

Considering the potential role of body image dissatisfaction and eating psychopathology during pregnancy, a systematic review and meta-analysis was conducted in order to investigate their prevalence and burden of eating psychopathology according to gestational age. Additionally, as a secondary objective, a systematic review was conducted to discuss the current literature on the role of body image and its interactions with psychosocial factors (please see the Materials and methods section for how thematic analysis was conducted).

## Materials and methods

The present systematic review and meta-analysis has been redacted in accordance with PRISMA 2020 guidelines [24]. In accordance with similar literature on the topic, postpartum was defined as a period of up to 1 year after delivery [7]. Inclusion criteria were as follows: the study investigated body image satisfaction, body image concerns or eating disturbances. Only studies enrolling at least one group of women during the peripartum were included, whether cross-sectional or longitudinal in design. The studies needed to be in English, and enrolled populations either being from the general population or individuals with a specific diagnosis of ED. Studies were excluded if the study included a population which underwent surgical complications during delivery or pregnancy. Moreover, studies were excluded in case of major psychiatric comorbidities (substance abuse, psychiatric diagnoses other than ED). Participants, Interventions, Comparators and Outcomes (PICO) criteria are reported in Table 1. This review’s protocol was pre-registered and is available at the stable link https://osf.io/qv3yt.

**Table 1:** Search strategy according to the population, intervention, comparison and outcomes (PICO) model

Parameter	Inclusion criteria	Exclusion criteria
Population	Women during pregnancy or postpartum (maximum 12 months) Either general population or eating disorders patients	DSM or ICD diagnosis other than eating disorders Report of major surgical or medical complications Overlapping population with more recent paper
Interventions	No intervention	Any surgical, medical or psychotherapy intervention during pregnancy
Comparison	Reported means and correlations in included population (cross-sectional or longitudinal studies) Differences between populations (case–control studies or randomized controlled trails)	NA
Outcomes	Psychometric evaluation of body image disturbances, body dissatisfaction and pathological eating behaviors	NA

### Information sources and search strategy

The authors used the electronic database PubMed in order to select studies, as the focus of the search was clinical in nature and this academic search system is the most extensive database for biomedical studies (> 29 million records, with the earliest record dated 1790 C.E.), as it allows the usage of boolean operators in order to optimize search strings, and as in contrast to other systems it is reported as reproducible in terms of identical results for repeated identical queries (e.g., subscription-based platforms, Google Scholar [25]). The following strings were used for the systematic search: *“("eating disorders" OR "eating psychopathology" OR "eating disorder" OR "anorexia" OR "bulimia" OR "body image" OR "body uneasiness")) AND ("pregnancy" OR "postpartum" OR "post-partum") NOT ("review"[ti] OR "case report")”.*

The terms included in the string were chosen to reach a broader scope over the scientific literature, irrespective of specific diagnostic criteria for eating disorders (as in, including “anorexia” rather than “anorexia nervosa”). PRISMA2020 was used in order to graphically represent the flow of information from the search to the final inclusion [26]. The last search was run in May 2023.

### Data selection process

L.T.; E.C.; P.G.M.; V.P; G.S independently assessed the abstracts of potentially eligible studies. Eligibility assessment was performed in an unblinded standardized manner. If there were doubts concerning the eligibility of the study for inclusion, the reviewers examined the full text of the articles. The published protocol required consensus in case the authors disagreed on the inclusion of a specific study. In case the opinion was not unanimous, a majority vote would have been taken between all authors. The authors agreed on all the eligibility assessments of the studies, and no consensus vote needed to take place. L.T.; E.C.; P.G.M.; V.P; G.S independently extracted several categories of data from each included study: questionnaire adopted, mean and standard deviation of each questionnaire regarding body image dissatisfaction or eating-related psychopathology, population enrolled. Only studies employing validated questionnaires were included. A threshold of a minimum of four studies (investigating the same construct with the same questionnaire) was selected for the meta-analysis. Studies were finally divided into thematic discussions [27]. L.T.; E.C.; P.G.M.; V.P; G.S. independently assessed the studies and proposed a number of themes in order to report a systematic overview of included studies. A majority vote between all authors was taken in order to choose the final five thematic categories:

- the influence of body image concerns on the adoption of breastfeeding;
- the interplay between body image dissatisfaction and peripartum depression;
- the sociocultural determinant of body image concerns in association with pregnancy;
- body image concerns and sexual disturbances during the peripartum;
- experiencing or reporting an altered food intake during pregnancy.

Studies including both the general population and eating disorder patients were evaluated for the meta-analysis and systematic review. As no study was found enrolling patients with eating disorders, and objectively investigating body image disturbances, and as a total of eight individual studies investigated pathological eating behaviors in the same population, but with an overall of less than three separate studies for each instrument, studies enrolling patients with eating disorders were not deemed eligible for the meta-analysis. In fact, out of the eight studies comprising a sample characterized by a clinical diagnosis of eating disorder, one employed the Eating Disorder Inventory 2 [28]; one the Eating Disorder Examination Questionnaire—36 items [29]; one administered the EDE-Q via telephone [30], two employed the EDE-Q—22 items [31, 32], two administered the EDE-Q with only a subset of items [33, 34], one study conducted a structured interview based on EDE [35].

Moreover, 14 studies utilized the Multidimensional Body-Self Relations Questionnaire Appearance Scales—MBSRQ in healthy pregnant women, and were also excluded for similar concerns [36]. These studies employed various versions of the scale: three studies used a 34-item version [37–39], one study used a 14-item version [40], another used a 46-item version [41], one study used a 68-item version [42], one study used a 69-item version [43], and two studies used a 7-item version [44, 45]. Furthermore, five studies utilized a 9-item version based on the BASS subscale, but data on mean sum scores and standard deviation was unavailable for two of these studies [46–50]. Overall, in order to reduce potential bias, a synthetic analysis through meta-analytic methods was not conducted, considering their methodological incongruence.

### Meta-analysis

A meta-analysis of included studies was performed in order to derive population means for each specific psychological construct of choice (body image dissatisfaction, eating behaviors). As multiple different questionnaires can be used for assessing these constructs, a population mean was extracted for each construct with at least four individual studies using the same questionnaire, in order to enhance the replicability, predictive power and reliability of the current analysis. As derived modified versions of a single questionnaire may exhibit divergent factorial validity or mean value, they were treated as a separate instance of a questionnaire [51]. Sub-group means according to trimester, postpartum period, or gestational age were derived in order to assess potential longitudinal trends. If one study had more than one clinical population enrolled and assessed in the protocol, and a mean value was offered, the data point for each was noted. As a large heterogeneity between studies was found (i.e., I2 > 25%), only random effects were described for each subpopulation, as well as their 95% confidence interval (C.I.) [52, 53]. Approximate means were estimated using the inverse variance method for pooling and weighting [54]. Standard normal distributions were used to transform individual means to Z-scores, in order to estimate 95% C.I. [54]. Between-study variance was calculated as described by Viechtbauer [55], through a restricted maximum-likelihood estimator. Forest plots were illustrated for each questionnaire and each psychological construct. All analyses were performed with R 4.3.1 [56], with the support of the following libraries: *meta* [54], *tidyverse* [57].

### Risk of bias

Five authors (L.T.; E.C.; P.G.M.; V.P; G.S) independently assessed the risk of bias for individual studies using the JBI critical appraisal checklist for cross-sectional or longitudinal observational studies [58], ROBINS-I for non-randomized interventional studies [59], RoB2 for randomized controlled trials [60]. In case the opinion was not unanimous, a majority vote would have been taken between all authors. These five authors agreed on all the eligibility assessments of the studies, and no consensus vote needed to take place. A figure representing the detailed assessment of the risk of bias for each article included in the review was given in the Supplementary Materials [61].

## Results

**Fig. 1:**
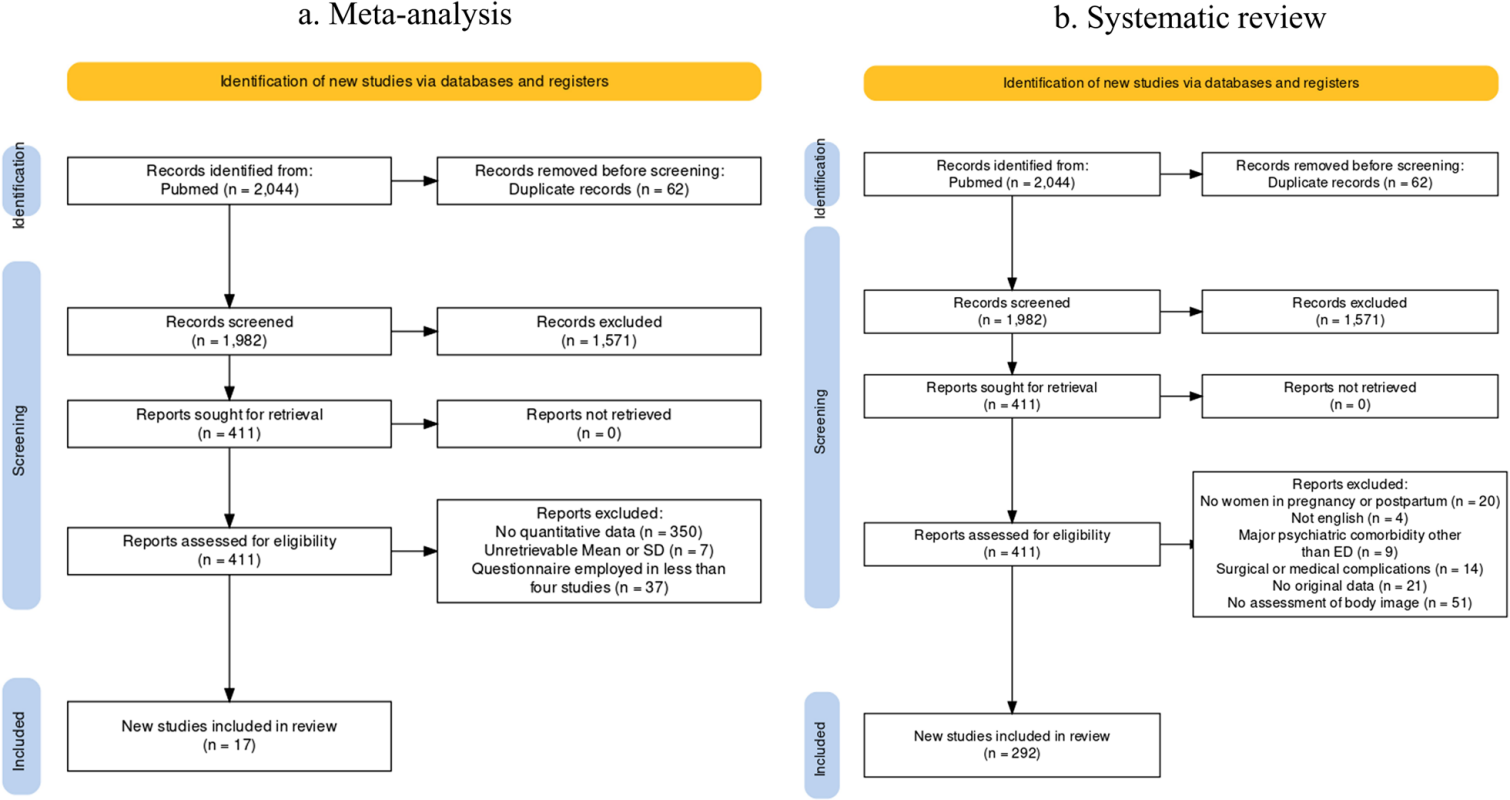
Flow diagram of the included studies. **a** Meta-analysis. **b** Systematic review

A total of 2044 studies were found through PubMed, and 62 duplicates (due to multiple indexing, updates or corrections to the original publication or separate publications of the same study in different outlets) were then removed. A total of 1571 studies were excluded on the basis of title and abstract, and 119 were excluded after manuscript review and application of inclusion criteria. Consequently, 292 studies were finally selected. A critical appraisal of each included study is offered in Figure S1 of the Supplementary Materials (Fig. 1).

In the meta-analysis, ten individual studies were found for the construct of body image dissatisfaction, and seven for eating psychopathology (as evaluated by the Eating Disorder Examination Questionnaire, EDE-Q). Out of the ten studies investigating body image dissatisfaction, five employed the Body Image Scale (BIS—1666 individuals; [62–66]; and five employed the Body Shape Questionnaire (BSQ—959 individuals; [62, 65, 67–69], for an overall of 2625 individuals. The seven studies investigating eating behaviors by EDE-Q enrolled a total population of 2551 individuals [12, 67, 70–74].

### Meta-analysis: body image dissatisfaction and pregnancy

**Fig. 2:**
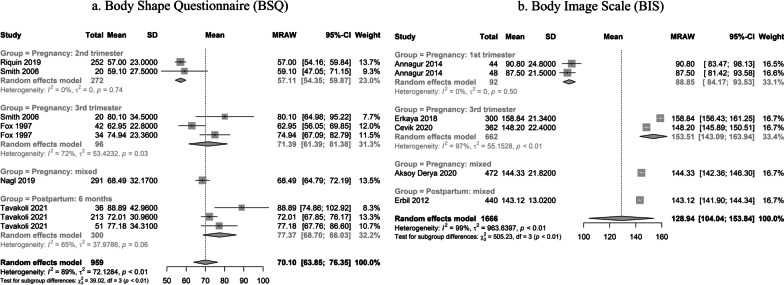
Body image dissatisfaction during pregnancy or postpartum. **a** Body Shape Questionnaire (BSQ). **b** Body Image Scale (BIS)

Five individual studies were found assessing body image dissatisfaction using the Body Shape Questionnaire (BSQ). Two studies enrolled more than one clinical group of women in the protocol: Fox et al. [75] enrolled both a sample of overweight women and a control group, while Tavakoli et al. [69] observed differences in body image satisfaction in association with breastfeeding practices (either adopting breastfeeding, bottle-feeding or a mix of both). Nagl et al. [71] enrolled pregnant women irrespective of gestational age and found a global mean by BSQ assimilable to those in the third trimester. Considering how the effect of the subgroup was significant for mean differences by gestational age (χ^2^ 39.02, *p* < 0.01), a trend was observed for higher body image dissatisfaction between the first and third trimester. No eligible study was found assessing body image satisfaction by BSQ in the 1st trimester, while a similar mean was found between the third trimester and the first 6 months of postpartum. Further details on single study means can be found in Fig. 2a.

Five individual studies were found assessing body image dissatisfaction by the Body Image Scale (BIS). One study, by Annagur et al. [63], enrolled both a group of women with hyperemesis gravidarum and controls, finding no significant difference in body image between groups. However, in full concordance with what was found in the current meta-analyses by BSQ, subgroups of gestational age or postpartum period were significant for mean differences (χ^2^ 505.23, *p* < 0.01). A similar trend for worse body image satisfaction was observed from first to third trimester, followed by a stabilization to-higher-than-baseline levels in the postpartum. Here, in contrast to BSQ, the only study on postpartum was on a population with up to 12 months of postpartum rather than 6 months. Further details on single study means can be found in Fig. 2b.

Therefore, the included studies suggest a trend for progressively worsened body image concerns from the first to the third trimester, and highlighted how body dissatisfaction might continue in the postpartum for at least 12 months after delivery.

### Meta-analysis: eating behaviors and pregnancy

**Fig. 3:**
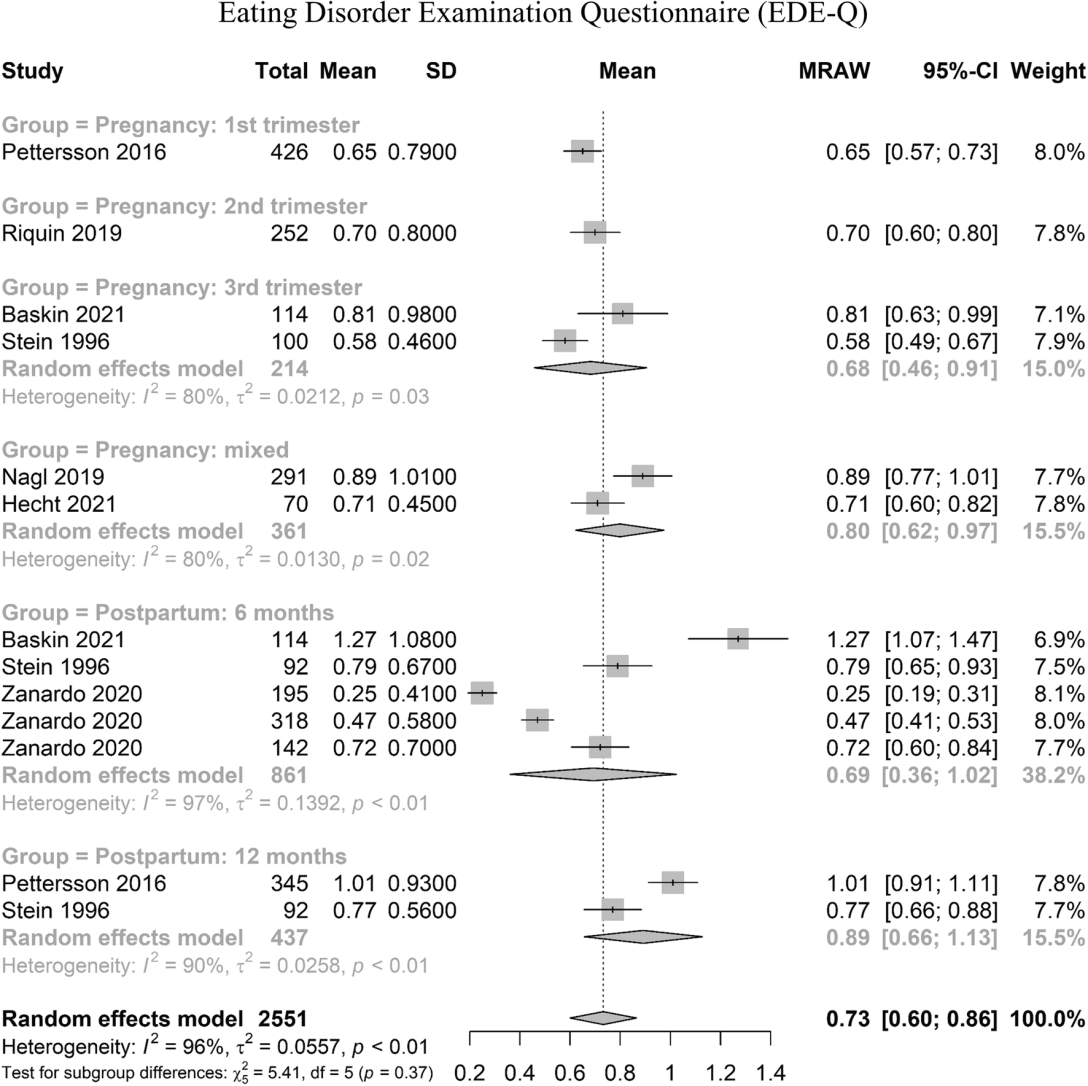
Eating behaviors during pregnancy or postpartum, by Eating Disorder Examination Questionnaire (EDE-Q)

Seven individual studies were found concerning eating behavior disturbances during pregnancy or up to 12 months after delivery. All seven included studies used EDE-Q. In contrast to body image dissatisfaction, no significant evidence was found for differences by gestational age in eating behaviors (χ^2^ 5.41, *p* 0.37). All gestational ages were covered by at least one study. Please see Fig. 3 for further details.

In summary, EDE-Q global means were comparable to those described in normative data for the general population [76, 77], signaling how pregnant women or women up to 6 months after delivery might not be interested in an aggravation of eating psychopathology in these time windows. By contrast, higher-than-baseline levels of eating psychopathology were found at 12 months in the postpartum.

### Breastfeeding and body image concerns

Among the general population, women who breastfeed until the child’s year of life showed more positive body images and were less likely to engage in maladaptive weight control behaviors than women who stopped breastfeeding early or had never breastfed their offspring [78]. Conversely, pregnant women who have greater body concerns are less likely to initiate breastfeeding [78], and the choice of bottle-feeding was associated with higher body dissatisfaction and higher fat intake [79–81]. Antenatal concerns such as embarrassment regarding public feeding and the impact of breastfeeding on breast shape were observed in correlation with a higher likelihood to adopt artificial milk, and body image dissatisfaction during pregnancy was associated with a shorter adoption of breast feeding practices [82]. Among women in postpartum, breastfeeding would seem to involve benefits on perceived body image correlated with increased awareness and appreciation of body functionality and fewer maladaptive weight control behaviors, as assessed by both psychometric tools and qualitative interviews (employing the Body Appreciation Scale—BAS, the Multidimensional Body-Self Relations Questionnaire Appearance Scales—MBSRQ, and the Eating Attitude Test [38, 78, 83]).

Partner influences were described to influence body image preoccupations, thin-ideal internalization and consequently breastfeeding self-efficacy [84, 85], which in turn has consistently reported predicting the likelihood of breastfeeding itself [86, 87]. The social discomfort of breastfeeding in public has been repeatedly reported as an inhibitor to its adoption, and obese women were significantly more likely to report social discomfort [88]. Nonetheless, contrasting evidence was present in the literature, with at least one study describing a higher likelihood of initiating breastfeeding when mothers experienced higher weight concerns [89], and a higher prevalence of body dissatisfaction and eating behaviors in obese mothers, who were in turn more likely to maintain exclusive breastfeeding or postpone weaning [90]. This finding might be interpreted in light of cultural factors, as contrasting evidence was offered by two studies conducted in Europe—Norway and Italy [89, 90]—in comparison to the rest of the world—predominantly English-speaking: United States, United Kingdom, Nigeria, and Australia [39, 79, 86–88, 91].

While mothers with a history of ED seem less likely to initiate breastfeeding [92], at age one, their infants had a higher diet quality in comparison to controls [76]. By contrast, socioeconomic factors such as ethnicity and income strata did significantly influence the fat intake of pregnant women, which might impact the quality of feeding for infants [77]. During postpartum, obese/overweight women reported a lack of body confidence more frequently than normal-weight women. Moreover, body dissatisfaction was observed as negatively associated with breastfeeding duration [92–94]. Therefore, a higher risk of early interruption of breastfeeding is observed in this group of women [95]. This is further supported by a study based on the Norwegian Mother and Child Cohort, reporting that women with anorexia nervosa and with an ED not otherwise specified (NOS)—purging subtype had an increased risk of early breastfeeding interruption [96]. In another qualitative study, most participants with a pre-existing ED reported severe distress regarding breastfeeding, and an urgency to interrupt breastfeeding in order to resume eating and compensatory behaviors [97]. Nonetheless, a social pressure to refrain from interruption was noted, as it was deemed contrary to the newly acquired ‘mother’ role. Moral judgments in this sample were predominantly negative on preferring one’s own weight preoccupations over the child’s needs [97].

### Body image dissatisfaction and peripartum depression

Considering the included studies pertaining to this topic, moderate support was found in favor of a role for depression in worsening body image during pregnancy—that is, women with depression had a higher likelihood of being unsatisfied with their body image [67, 98–105]. This relationship was also true for the postpartum [106, 107]. Importantly, the association between body image dissatisfaction and postpartum depression seems to be transcultural [102, 106, 108–115]. Contrasting evidence, however, was offered by three studies, which found no relationship between these body image concerns and depressive symptoms (as assessed by the Center for Epidemiological Studies Depression Scale—CESD, the Patient Health Questionnaire—PHQ, the Depression Anxiety Stress Scales—DASS, the Body Cathexis Scale—BCS, the Body Shape Questionnaire—BSQ, and the Ben-Tovim–Walker Body Attitudes Questionnaire—BAQ [93, 116, 117]).

Depression is also linked to a premature interruption of breastfeeding in postpartum, possibly through the mediation of body image concerns [33]. Several other factors were observed concurrent to the relationship between body image dissatisfaction and postpartum depression. For instance, women with low self-compassion exhibit worse satisfaction with their body image and also higher depressive, anxiety and eating symptoms [91, 118–122]. Body shaming mediated the relationship between body dissatisfaction and depression [123]. Similarly, partner support seems to protect against depression during or after pregnancy [124, 125]. Low-income women in postpartum were observed as exhibiting worse psychosocial and behavioral health in multiple domains, including depression and body image dissatisfaction [114]. A higher likelihood of retaining excessive weight from pregnancy was associated with depressive symptoms and body image dissatisfaction during or after pregnancy [126–129], thus offering evidence in favor of an interplay between eating behaviors, body image concerns and depression in this population. While the majority of the scientific literature established body image dissatisfaction as a causal factor in postpartum depression [46, 49, 50, 102, 109, 130–139], the opposite was also suggested [131], and at the present moment no definite direction of causation can be derived. Importantly, the association might also be considered bi-directional [140].

For what concerns women with a diagnosis of EDs, this population showed greater depressive symptoms scores than women with no history of an ED [35, 141–146]. The incidence of binge eating disorder was significantly associated with major depressive symptoms in pregnancy compared to other EDs [147, 148]. Additionally, depressive symptoms were found to increase the rate of transition between EDs [149]. Contrasting evidence was also present in the literature on the topic for what concerns women with an ED, with one study finding no difference between gestational and postpartum levels of depression in these women [150]. However, depression seems to be correlated to emotional eating and restrained eating attitudes [151], while it seems that there is no correlation between depression symptoms and relapse of eating disorders during postpartum [152]. Finally, weight stigma was found to worsen depressive symptoms in pregnant women [153].

### Sociocultural influences and ethnicity

Socioeconomic factors such as ethnicity and income seem to significantly influence both food intake and body image perception in pregnant women [154]. In fact, a number of studies highlighted how low-income and ethnic minority statuses were positively associated with distress, ED symptoms and depressed mood [64, 93, 148, 155, 156]. Other studies highlighted how, in the USA context, ethnicity was partially decoupled from income. African-American women, in fact, seemed to have less severe body dissatisfaction during and after pregnancy [157, 158].

Adolescent pregnant women, who are transitioning towards parenthood, showed lower maternal adjustment, poorer maternal attitudes, more negative attitudes to sex and worse body image in comparison to adult pregnant women, even when other confounding factors are considered (such as socioeconomic status; [159]. Similarly, unemployed and pregnant women without a partner exhibited worse body image preoccupations in comparison to their peers [159].

Three studies investigated the effect of magazines primarily dedicated to pregnant women [160–162]. The main topics were dieting, weight loss, and physical exercise. In these studies, media outlets on average worsened the subjects’ body images and mood levels. The representation of pregnancy was often described as unrealistic, hiding the physiological modifications of gestation. In fact, the media often ignored common bodily experiences such as acne, stretch marks or asymmetric abdomens. Traditional media also emphasized that it is important to “bounce back with the same effort one would employ when recovering from an illness” and thus return to the pre-pregnancy weight as soon as possible [163]. Online content on social networks seems to replicate similar concerns [164–167]. Similarly, two studies found that the amount of time spent on Facebook correlated with body dissatisfaction during pregnancy [168] and with ED psychopathological dimensions in the postpartum period [169]. Weight and obesity stigmatization during pregnancy, assessed through direct means such as validated questionnaires (as the Perceived Sociocultural Pressure Scale—PSPS, and the Fat Talk Questionnaire—FTQ), interviews, and indirect instruments (as social network sentiment analysis), was also found to correlate with eating disorder psychopathology, body dissatisfaction and depressive symptoms [123, 170, 171].

Another line of research investigated the impact of close relationships on body image during the peripartum, in particular for what concerns romantic partners. The influence of the partner’s judgment on attractiveness was described as high and bi-directional: both as a reassuring or supportive factor [43, 69, 172–175], and also as a source of distress [86, 125, 148, 153, 176]. The degree of influence for the partner was greater when the relationship was clearly dysfunctional (e.g., substance addiction, intimate violence towards the pregnant woman; [155, 177], possibly due to relative social isolation and lack of social support. In fact, it appears that during pregnancy the need for social support and reassurance increases compared to the general population. The explanations for this effect are likely both cultural and psychological, driven by biological factors [178].

A similar pattern of “dual” potential effects also emerged regarding the role of healthcare professionals (e.g., midwives, obstetricians, gynecologists, allied health professionals). Despite sometimes being given positive feedback and also despite being described as helpful and supportive at times [87, 102], in most studies healthcare professionals were criticized for exhibiting inefficient communication skills [179, 180]. For instance, one paper highlighted how EDs among pregnant women were under-investigated by medical personnel [181]. In another, healthcare professionals were criticized for judgmental attitudes towards obese pregnant women, with an impact on their body image [182]. On the other hand, in two cases, pregnancy in the psychodynamic setting was used by the therapist as an effective means in order to approach the difficult theme of femininity and body image [183, 184].

In brief, among studies that touched on the topic of mother–child relationship, the majority of women positively described their newfound motherhood and claimed that it had improved their self-esteem and sense of fulfillment [87, 155, 174], despite the child being in some cases a source of performance anxiety—i.e., not being a good mother [140, 185]. In a relative minority of studies, though, the child and the burden of peripartum were underlined, especially regarding the reduction in social activities outside of the child and the perceived reduction in attractiveness [101, 186–188]. One of the studies also highlighted a particular issue, the socially induced guilt of “not being happy” despite the experience of motherhood among women with postpartum depression [101].

Most of the aforementioned studies took place in Western countries; it is thus necessary to evaluate the sociocultural determinants of bodily preoccupations during pregnancy [189, 190]. In traditional societies, mothers are generally expected (and often report self-appreciation) to exert a full-time parenting role, with associated ideal qualities of “motherhood”. In turn, adherence to the “mother-hood” ideal may be correlated to a solid social support net [111, 174, 188, 191]. Conversely, stronger gendered ideals have also been associated with a higher drive for thinness, especially in Japan [45, 192].

### Sexuality, body image concerns and pregnancy

Qualitative studies highlighted a relationship between body image concerns and sexual functioning [42, 140, 141, 172, 185, 186, 193, 194]. This evidence is replicated in studies employing objective psychometric measurements [62, 172, 195, 196]. Up to 61% of women expressed a concern for the lower attractiveness to partners, and those who felt physically appreciated described their identity as sexual beings as intact [172]. To be noted, an early study conducted in 1986 with preliminary evidence of lower desire, higher frequency of sexual dysfunctions and higher bodily dissatisfaction during postpartum [197]. Body image concerns did in part explain the degree of sexual dysfunctions experienced during the peripartum, as assessed by the Body Image Scale (BIS) and Female Sexual Function Index (FSFI). This relationship remained significant after controlling for the confounding variables: education, living place, trimester of pregnancy, number of pregnancies [62], working status, spontaneous or induced interruption of pregnancy, comorbidity [198]. A theoretical model supported by objective measurements suggested that the relationship between body image and sexual functioning may actually be fully mediated by the degree of cognitive distraction with the appearance of the body during intercourse [199]. In a large sample of women enrolled during prenatal educational classes in Turkey (472 participants), up to 50% of women experienced sexual dysfunction during pregnancy [62]. This high prevalence seemed to be replicated in a sample of pregnant women enrolled in Iran, where out of 206 participants 72.3% of women reported a high score when assessed by FSFI, below or equal to 28 [41]. However, contrasting evidence was offered, and other studies reported a non-significant relationship between these two constructs while describing a relationship between the subjectively perceived weight and sexual satisfaction or capacity to reach orgasm [41]. Similarly, relationship factors (such as communication) appeared to be more correlated with sexual satisfaction during the third trimester than body image self-appreciation [40].

For what concerns postpartum, studies reported a higher likelihood of sexual dysfunction after giving birth as a function of body satisfaction and body self-consciousness during physical intimacy [194, 200, 201]. In fact, a hypothesized model showed a significant relationship between neuroticism and sexual functioning, as mediated by body satisfaction and anxiety about body exposure during sexual activity [201]. A significant contribution to sexual functioning in postpartum women was described for genital image concerns [133, 202, 203], for which mode of delivery had a significant difference (vaginal delivery as compared with cesarean section; vaginal delivery correlated with worse genital self-image; [200]. Additionally, breastfeeding and pre-existing dyspareunia were associated with significant sexual dysfunctions at 6 months postpartum [204]. Body image during postpartum, in relation to sexual functioning, was a concern in approximately 50% of mothers interviewed, but also 40% of fathers [205]. Both at 4 and 12 months postpartum, mothers reported an unresolved change in sexual self-perception [205, 206].

### Food intake: perception and reporting during pregnancy

Included studies highlighted a higher risk of an altered food intake perception or report during pregnancy [207–210]. In fact, misreporting of energy intake was observed among overweight or obese pregnant women in a study conducted in Australia, with up to a third of the sample under-reporting their energy intake [208]. Adequate reporting of energy intake was assessed by estimating the ratio between the reported intake and basal metabolic rate. Interestingly, the prevalence of under-reporting was higher at 36 weeks of pregnancy in comparison to early pregnancy (10–20 weeks of gestation). Women who under-reported energy intake were more likely to have a higher discrepancy score between perceived and pursued body shape (at 36 weeks of gestation [208]). Moreover, women who under-reported energy intake were more likely to have a history of multiple previous dieting attempts, but less likely to report body dissatisfaction at either 36 weeks or 10–20 weeks of gestation [208]. Under-reporters were also more likely to be at risk for depression [208]. This evidence thus suggests that weight and shape concerns, as well as pathological eating behaviors, might be aggravated in predisposed individuals during pregnancy.

Similarly to what was previously found in the general population of non-pregnant women, early unwanted sexual experiences were associated with both eating problems and compensatory behaviors during pregnancy, as well as with a marked concern for shape and weight [211]. Known risk factors for eating disorders thus seem to apply also during pregnancy or postpartum. Among women with severe mental illness, a high rate of obesity and low adherence to serving recommendations during pregnancy were described [207]. Up to 19% of pregnant women diagnosed with a severe mental disorder were identified with a potential ED [207]. Sugar and processed foods seem to be consumed at a higher than recommended level among this population, and food cravings or psychological distress were among the main perceived barriers to nutritional wellbeing [207]. Among facilitators of adherence, access to a dietitian, correct information delivery and support, and comprehensive care were found to facilitate nutritional wellbeing in severely mentally ill pregnant women [207].

Moreover, for what concerns EDs, a large sample conducted in Norway provided evidence that women with a past or present history of binge eating had a higher intake of total energy during pregnancy, as assessed by a semiquantitative self-administered food questionnaire [210]. Nonetheless, depressive symptoms and eating behaviors were not associated with a higher likelihood of engaging in physical activities [212]. Indeed, eating psychopathology was associated with lower total energy expenditure during pregnancy, suggesting either a higher resistance to engage in compensatory behaviors or a higher incidence of binge eating rather than anorexia or bulimia nervosa in the evaluated samples [212].

Finally, as evidence for the transcultural dimension of this phenomenon, in a study conducted in Japan up to 12.9% of women were identified as under-reporting food intake. This evidence was objective, as women were assessed by comparing objective urinary excretion levels and self-administered food intake questionnaires. Under-reporters had a lower pre-pregnancy body weight index and lower gestational weight gain, while also reporting a higher desire to return to pre-pregnancy weight soon after childbirth [209].

## Discussion

The present meta-analysis and systematic review aimed at evaluating the prevalence and longitudinal trends of body dissatisfaction and eating behaviors during pregnancy. Body dissatisfaction was found to be progressively elevated during pregnancy, and to remain at higher-than-baseline levels in the postpartum period (up to 12 months after delivery). By contrast, EDE-Q scores among pregnant women were comparable to those described in normative data for the general population [76, 77]. In comparison to other reviews or meta-analysis [213, 214], no higher risk of eating behaviors during pregnancy was found, and no longitudinal trend by gestational age was noted for eating disturbances. However, higher-than-baseline eating behaviors disturbances were found at 12 months in the postpartum.

The included studies’ quality, as assessed by the chosen risk of bias evaluation tools, was satisfactory except for the following categories: the incomplete follow-up management in longitudinal studies, researcher’s own influence on the study among qualitative studies and the randomization process among randomized controlled studies (more than 50% of the studies were classified as “High Risk of Bias” in these categories). While the overall quality of the studies was acceptable, future research can address these specific limitations in the current scientific literature. Other details regarding the assessment for the risk of bias can be found in the Supplementary Materials as Figure S1.

### Body image dissatisfaction and pregnancy

Similar to what was previously described in the general population for non-pregnant women [215], a high degree of diagnostic crossover was observed during pregnancy [210]. In fact, in a well-powered Norwegian cohort study, out of 213 women diagnosed with BN before pregnancy, 34.27% were diagnosed with BED and 34.27% recovered during pregnancy. Similarly, out of 1267 women diagnosed with BED before pregnancy, 38.51% recovered during pregnancy [210]. These studies highlight the importance of assessing individuals in a longitudinal manner, as the degree of diagnostic crossover may impede a direct correspondence between diagnosis, outcomes, and psychopathological scores, while also suggesting pregnancy as a critical time window during which women might experience bodily distress, and thus reshape eating behaviors and the relationship with their own body.

Body image concerns and dissatisfaction, as investigated by two different instruments (BIS, BSQ), showed convergent evidence for a trend of progressively worse satisfaction and elevated body image concerns between the first and third trimester. Convergent evidence was also found for a relative period of stability on higher-than-baseline levels for the postpartum, both at 6 and 12 months after delivery. Therefore, the present meta-analysis supports the hypothesis that the increased risk for higher body image dissatisfaction in association with pregnancy may not resolve even after 12 months postpartum [205]. This finding revisits and expands previous works on the topic and may update both clinical practices and future research [208, 212], suggesting that psychological evaluation(s) before 12 months in postpartum might be a potentially impactful health policy in order to detect body image-associated psychopathology. In order to confirm this finding, more research on the subject is nevertheless warranted.

The importance of body image concerns as driven by social pressures, informing breastfeeding practices and impacting on sexuality was critically assessed, and here briefly discussed in light of other theoretical or empirical considerations. In summary, the authors notice the need for a diagnostic tool capable of identifying and screening those women at a high risk of either developing body image dissatisfaction during pregnancy, or already experiencing body image concerns before pregnancy, in order to develop and offer targeted interventions. The current literature has not yet derived a clear direction of causality between body image concerns and depressive symptoms. Nonetheless, early detection of signs and symptoms of depression seems warranted, in particular for women with a past or current diagnosis of an eating disorder in this clinical population, in order to alleviate the burden of pathological eating behaviors during pregnancy.

Breastfeeding was associated with lower body image self-consciousness during physical intimacy in postpartum women [200], which in turn may result in higher sexual functioning [201]. As breastfeeding is warranted by current guidelines [216, 217], general practitioners, gynecologists, pediatricians and mental health professionals should promote higher adoption of breastfeeding in pregnant women. Health professionals should identify those pregnant women at higher risk for body image dissatisfaction, for which social exposure while breastfeeding may present a significant source of distress. As the current systematic review highlighted a potential causal link between body image dissatisfaction and sexual dysfunctions, greater attention to addressing body concerns seems warranted for what concerns women undergoing pregnancy.

### Eating behaviors during pregnancy

A diagnostic tool capable of identifying and screening those women at high risk for eating psychopathology during pregnancy seems to be warranted, even if eating behaviors may not concern as diffusively and pervasively pregnant women as body image dissatisfaction. A recently developed tool for this purpose is the Prenatal Eating Behaviors Screening tool, which seems to have satisfactory sensitivity and specificity, while being easy to administer and only 12-item long [218].

Altered food intake perception or report during pregnancy might also drive eating behaviors. Two factors might influence an altered subjective perception or objective report of food intake during pregnancy, namely sociocultural pressures or body image concerns [219]. In fact, women might experience pressure or guilt in relation to optimal nutritional adherence during pregnancy, but also a heightened awareness of body shape and weight [219–221]. However, the change in the perceived function of a pregnant body as an object for others, through the gaze or intent of the others [222, 223], might shift the focus from esthetic to functional concerns [172]. In other words, the body might be lived and interpreted from an object judged by beauty standards to an object with the primary aim of nurturing (a) child(ren). Therefore, a reduction in body image concerns during pregnancy might be expected if societal pressures shift the balance towards an object lived as a function of fertility and nurturing. In fact, attitudes towards weight gain during pregnancy were observed as being associated with both a reformulation of a positive body image and family support during gestation [224], especially for adolescents [129]. Indeed, objective studies confirm that in healthy women, not characterized by body image concerns or eating psychopathology, body shape assessment during pregnancy can be adequate, dietary restrictions may not be attempted, and larger body sizes might be preferred in comparison to ideal physical appearances desired before pregnancy [172, 225, 226].

Across the population, food intake is consistently associated with under-reporting when assessed subjectively and by memory recall (or through the use of eating diaries; [227]. This distinction seems to pertain both to overweight or obese individuals who under-report as a consequence of shame, guilt, or lack of concern [208] but also, by contrast, to individuals with a high degree of body dissatisfaction and a high interest in enduring true dietary restrictions [209, 227]. For these reasons, access to a dietitian seems to be warranted for pregnant women [207]. While guidelines have not been currently developed on the topic, priority should be reserved for those individuals with a high tendency for under-reporting and for those with a previous history of multiple dieting attempts or eating behaviors. In order to detect true under-reporting, all allied health professions should collaborate in favor of comprehensive care.

### Strengths and limits

The current review employed random effects models to results presented in the scientific literature. This approach proved more appropriate for explaining the heterogeneity between studies. This result indicates that other significant influences—rather than gestational age alone—may explain the difference in mean values between included studies. Future research might then aim at explaining the residual variance observed, informed by the evidence gathered by the current systematic review over the role of relationship support, cultural factors, social (such as attitudes towards body image and pregnancy by healthcare providers) and demographic determinants.

As significant differences by trimester were found for what concerns body image preoccupations, future studies might also adopt a design apt to evaluate this determinant, avoiding collecting a single time point in time during pregnancy or comparing pregnant vs general populations irrespective of gestational age. Even if no similar trend was noted for eating behaviors, the authors warrant a similar design also in these studies, as the lack of current evidence might be due to under-representation in scholarly literature. Additionally, future research might focus on the longitudinal evaluation of individuals with eating disorders during pregnancy, in order to better understand changes in this clinical population. Finally, a higher harmonization in the research practice during pregnancy is warranted, possibly adopting a standard validated questionnaire, without deriving edited versions in order to enhance replicability and comparisons. The current review formally addresses this limitation in the scientific literature on the topic, allowing future researchers to properly estimate the cost/benefit to employ less common instruments in order to evaluate body image dissatisfaction or pathological eating behaviors in the peripartum. Namely, these less commonly used instruments were: the Amputee Body Image Scale (ABIS), Ben-Tovim Walker Body Attitudes Questionnaire (BAQ), Body Cathexis Scale (BCS), Body Image in Pregnancy Scale (BIPS), Eating Disorder Inventories—version 1 and 2 (EDI) and the Multidimensional Body-Self Relations Questionnaire (MBSRQ).

The current systematic review included qualitative studies. As already known, this particular type of study might be influenced by the researchers’ own views and culture [228], which were however seldom stated in the included studies (please see Figure S1c in the Supplementary Materials). This concern might be relevant in light of the geographical specificity of most studies here evaluated, where specific countries were over-represented (please see Table 2).

**Table 2:** Included studies, thematic grouping and related information

Theme	Study design	Enrolled population (total)	Represented countries
Body image concerns or pathological eating behaviors during pregnancy or postpartum (*N* = 292)	Case–control: 25 Cross-sectional: 154 Longitudinal: 78 Qualitative: 30 Randomized Controlled Trial: 5	General population: 551.144 Eating disorders: 145.322 (of which, 96 only AN, 204 only BN, the rest mixed diagnoses)	USA: 85 Australia: 37 UK: 29 Norway: 12 Turkey: 12 Other: 117
Body image concerns and breastfeeding (*N* = 16)	Case–control: 2 Cross-sectional: 8 Longitudinal: 5 Qualitative: 1 Randomized Controlled Trial: 0	General population: 3.375 Eating disorders: 6.196 (all mixed diagnoses)	USA: 9 UK: 3 Italy: 2 Netherlands: 1 Taiwan: 1
Body image dissatisfaction and peripartum depression (*N* = 42)	Case–control: 2 Cross-sectional: 20 Longitudinal: 18 Qualitative: 2 Randomized Controlled Trial: 0	General population: 154.280 Eating disorders: 165 (of which 43 only BN)	USA: 13 Australia: 8 UK: 3 Italy: 2 Norway: 2 Taiwan: 2 Other: 12
Sociocultural determinant of body image concerns during pregnancy (*N* = 25)	Case–control: 1 Cross-sectional: 11 Longitudinal: 4 Qualitative: 7 Randomized Controlled Trial: 2	General population: 49.247 Eating disorders: 0	USA: 13 UK: 3 Australia: 2 Other: 7
Body image concerns and sexual disturbances during the peripartum (*N* = 23)	Case–control: 2 Cross-sectional: 14 Longitudinal: 2 Qualitative: 3 Randomized Controlled Trial: 2	General population: 50.673 Eating disorders: 43 (all enrolling only BN)	USA: 6 Iran: 4 Turkey: 4 Portugal: 2 Taiwan: 2 Other: 5
Experiencing or reporting an altered food intake during pregnancy (*N* = 6)	Case–control: 0 Cross-sectional: 5 Longitudinal: 1 Qualitative: 0 Randomized Controlled Trial: 0	General population: 33.433 Eating disorders: 0	Australia: 3 Japan: 1 Norway: 1 USA: 1

AN: anorexia nervosa; BN: bulimia nervosa

The present systematic review was performed through an inclusive string search, but only by a single research bank, namely PubMed. However, by the number of assessed and included studies, the current work achieved sufficient coverage of the scientific literature on the topic. In the current review, the inclusion of papers was restricted solely to those published in peer-reviewed journals (thus excluding gray literature). Because of this, a potential selection bias could inflate estimated prevalences and mean scores at psychometric questionnaires. Finally, studies comprising a sample with major psychiatric comorbidities were excluded. It is reasonable to expect a significant influence of psychiatric comorbidities on both constructs under evaluation, but this endeavor was deemed beyond the scope of the current work, which was to derive normative values for the general population and investigate the spectrum of a continuum with eating disorders. The relatively low rate of inclusion (please see Fig. 1 in Results) could bias the overall estimates in the meta-analysis. A large number of thematically appropriate studies had to be excluded because of methodological issues. The most frequent cause of exclusion was the employment of incomplete or modified versions of otherwise validated questionnaires, which hinders the aggregation of results in a meta-analysis. For these reasons, in order to improve the replicability and generalizability of future studies, an overview of the most used instruments was reported in the Results.

Another limitation among the literature is the relative absence of studies on body image and eating psychopathology in pre-gestational studies (for example, with a longitudinal design). Similarly, no study was found comparing a population of pregnant women to the general population for what concerns determinants of sexual distress in relation to body image or eating concerns. Even though, as stated beforehand, normative scores for non-pregnant women regarding eating psychopathology were found as similar as those described among pregnant women, the hypothesis that eating psychopathology might manifest before (as in, among women seeking pregnancy) or during the first trimester cannot be completely ruled out.

### What is already known on this subject?

Women during pregnancy or in the postpartum are known to undergo a particular risk during this window of time. Nonetheless, no definite evidence on how peripartum might influence body image concerns or pathological eating behavior is available in the scientific literature at the moment.

### What this study adds?

The present review addresses the lack of scientific consensus on the topic of peripartum, body image dissatisfaction and pathological eating behaviors. It offers evidence in favor of progressively increasing body dissatisfaction by gestational age, and in the postpartum. Conversely, it fails to find statistically significant evidence of an increase in pathological eating behaviors in the same time period.

## Conclusions

Progressively higher body image dissatisfaction was observed during pregnancy, stably elevated for at least 12 months postpartum. No similar trend was observed for eating psychopathology, but a worse status at 12 months in the postpartum was observed. Moreover, the present meta-analysis offers normative values of body image satisfaction and eating-related psychopathology by gestational age. This evidence may inform future research on these topics and guide clinical practice by focusing on the subjective experience of pregnant women, in light of a comprehensive concept of healthcare, warranting careful history taking, in order to better evaluate individual risk factors (personal or relational), and to monitor longitudinal trajectories by gestational age and during postpartum.

## Supplementary Information

Below is the link to the electronic supplementary material.

Supplementary file1 (DOCX 500 KB)

## Data Availability

The database of the studies, with the extracted data items, can be shared upon reasonable request to the corresponding author.
