# Supplementary material for: Pregorexia: a systematic review and meta-analysis on the constructs of body image dissatisfaction and eating disturbances by gestational age in the peripartum
Source: Eat Weight Disord. 2023 Aug 1;28(1):64. doi: 10.1007/s40519-023-01595-8 (PMC10393903; doi:10.1007/s40519-023-01595-8)

# Supplementary Materials

*Figure S1a - Risk of Bias for cross-sectional studies, Joanna Briggs Institute Critical Appraisal Tools*


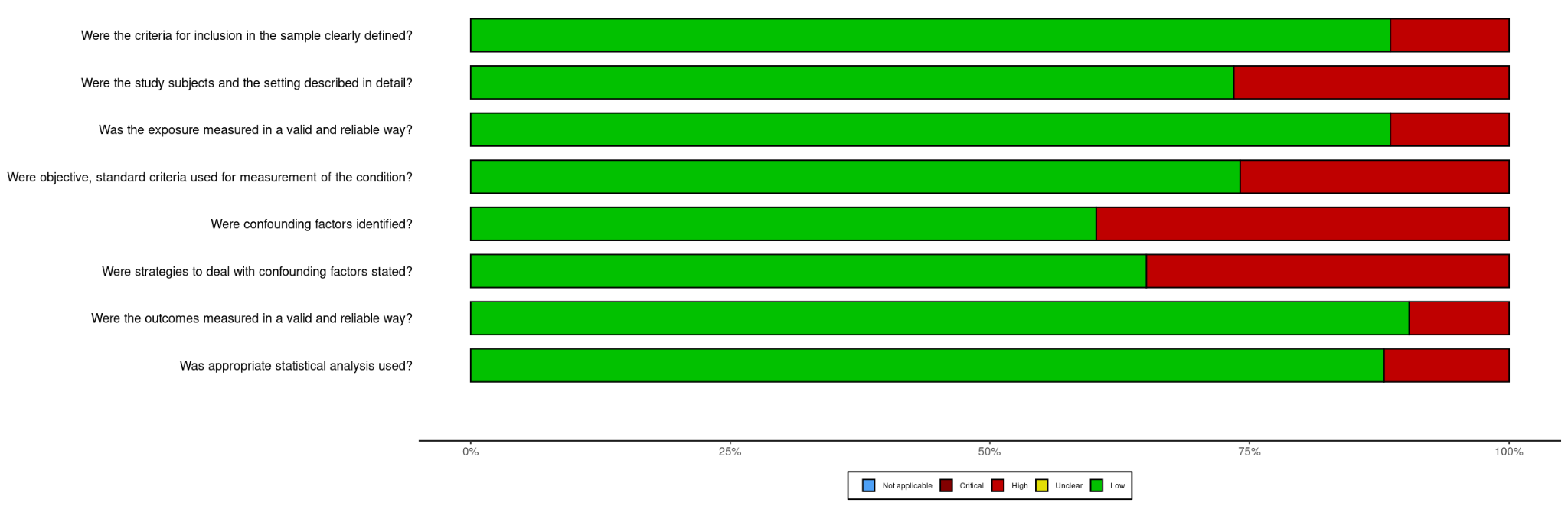


*Figure S1b - Risk of Bias for longitudinal studies, Joanna Briggs Institute Critical Appraisal Tools*

*
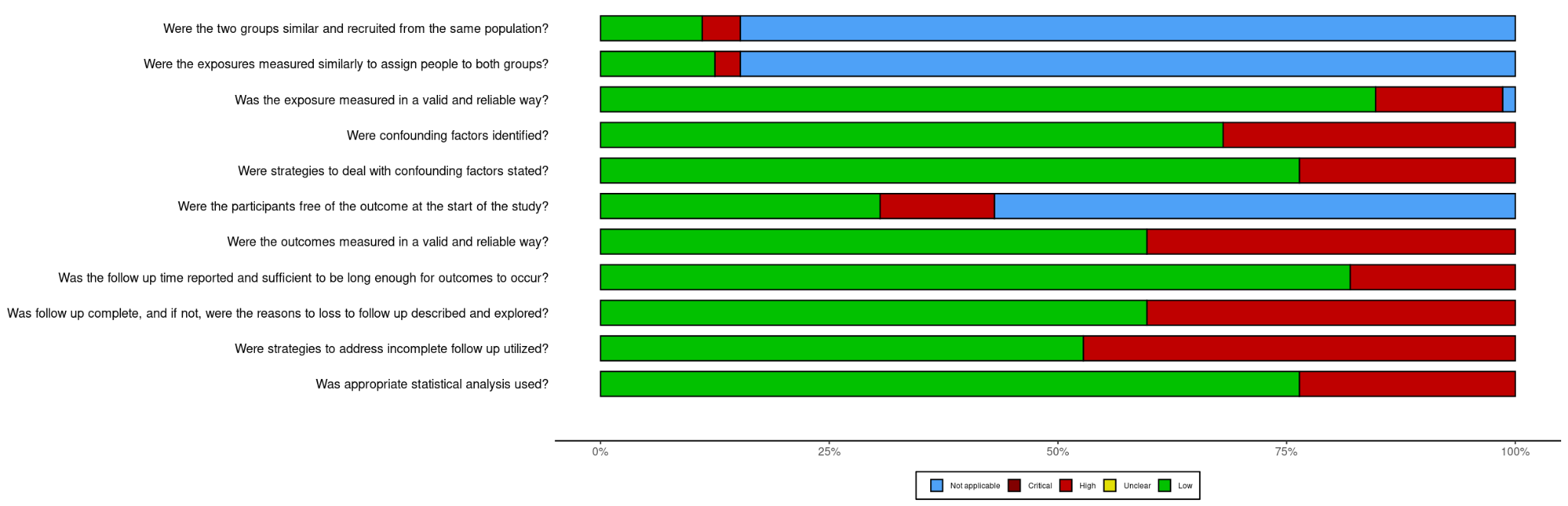
*

*Figure S1c - Risk of Bias for qualitative studies, Joanna Briggs Institute Critical Appraisal Tools*


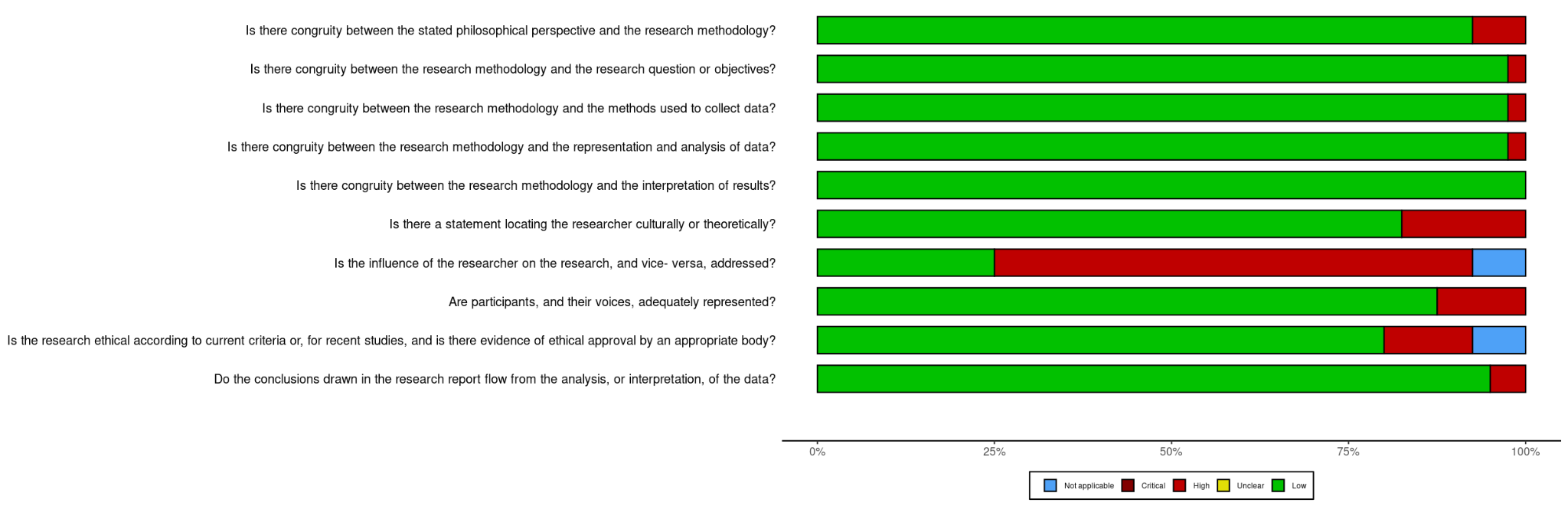


*Figure S1d - Risk of Bias for randomized controlled studies, Cochrane Risk of Bias 2*


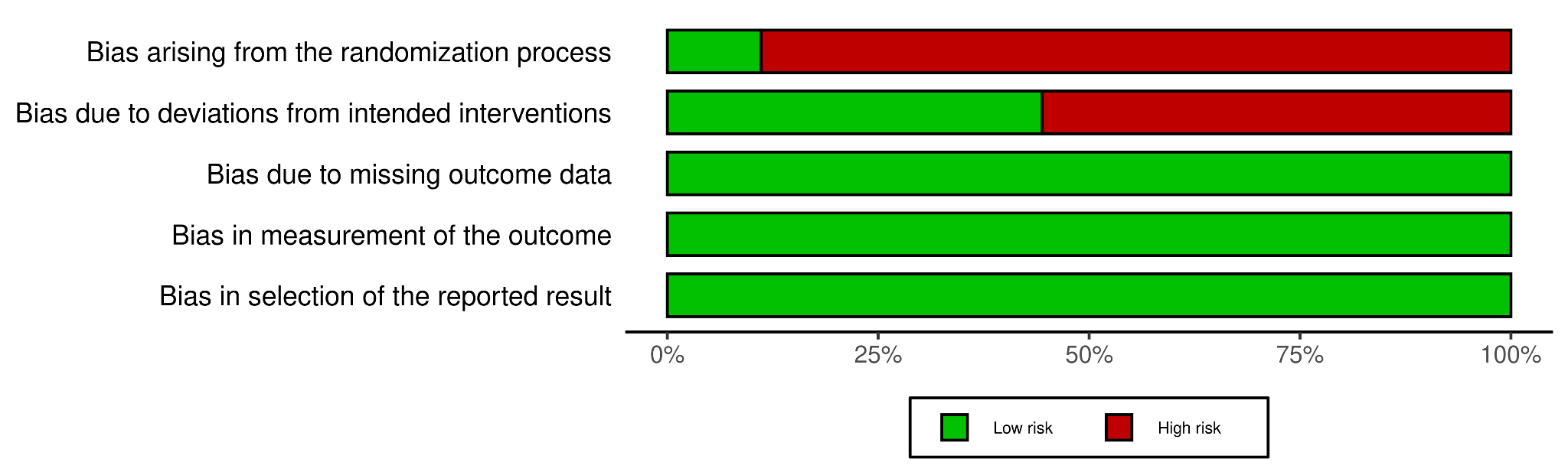

Supplement: Supplementary file 1 — Supplementary file1 (DOCX 500 KB) [file 40519_2023_1595_MOESM1_ESM.docx]
